# Comprehensive Evaluation of Pediatric Patients with Ebstein Anomaly Requires Both Echocardiography and Cardiac Magnetic Resonance Imaging

**DOI:** 10.1007/s00246-022-02948-3

**Published:** 2022-06-21

**Authors:** Lianne M. Geerdink, Wouter M. van Everdingen, Irene M. Kuipers, Zina Fejzic, Gideon J. du Marchie Sarvaas, Stefan Frerich, Henriëtte ter Heide, Willem A. Helbing, Chris L. de Korte, Jesse Habets, Livia Kapusta

**Affiliations:** 1 Department of Pediatric Cardiology, Amalia Children’s Hospital, University Medical Center Nijmegen, Nijmegen, 6525GA The Netherlands; 2 Department of Pediatric Cardiology, Wilhelmina Children’s Hospital, University Medical Center Utrecht, Utrecht, 3584EA The Netherlands; 3 Department of Medical Imaging, University Medical Center Nijmegen, Geert Grooteplein Zuid 10, Nijmegen, 6525GA The Netherlands; 4 Department of Pediatric Cardiology, Amsterdam University Medical Center, Amsterdam, 1105AZ The Netherlands; 5 Center for Congenital Heart Diseases, University Medical Center Groningen, Groningen, 9713GZ The Netherlands; 6 Department of Pediatric Cardiology, Maastricht University Medical Center, Maastricht, 6229HX The Netherlands; 7 Department of Pediatric Cardiology, Sophia Children’s Hospital, Erasmus Medical Center, Rotterdam, 3015GD The Netherlands; 8 Medical Ultrasound Imaging Center, Department of Medical Imaging, University Medical Center Nijmegen, Nijmegen, 6525GA The Netherlands; 9 Pediatric Cardiology Unit, Department of Pediatrics, Dana-Dwek Children’s Hospital, Tel Aviv Sourasky Medical Center, Tel Aviv University, 6423906 Tel Aviv, Israel

**Keywords:** Congenital heart disease, Ebstein anomaly, Pediatric, Imaging, Echocardiography, Cardiac magnetic resonance imaging

## Abstract

With the trend towards childhood surgery in patients with Ebstein anomaly (EA), thorough imaging is crucial for patient selection. This study aimed to assess biventricular function by echocardiography and cardiac magnetic resonance (CMR) and compare EA severity classifications. Twenty-three patients (8–17 years) underwent echocardiography and CMR. Echocardiographic parameters included tricuspid annular plane systolic excursions (TAPSE), fractional area change of the functional right ventricle (fRV-FAC), fRV free wall peak systolic myocardial velocity (fRVs’), and tricuspid regurgitation (TR). End-diastolic and end-systolic volume (EDV resp. ESV), fRV- and LV ejection fraction (EF) and TR were obtained by CMR. EA severity classifications included displacement index, Celermajer index and the total-right/left-volume index. Median fRV-FAC was 38% (IQR 33–42). TAPSE and fRVs’ were reduced in 39% and 75% of the patients, respectively. Echocardiographic TR was visually graded as mild, moderate, or severe in nine, six and eight patients, respectively. By CMR, median fRVEF was 49% (IQR 36–58) and TR was graded as mild, moderate, or severe in nine, twelve and two patients, respectively. In 70% of cases, fRV-EDV was higher than LV-EDV. LVEF was decreased in 17 cases (74%). There was excellent correlation between echocardiography-derived fRV-FAC and CMR-derived fRVEF (rho = 0.812, *p* < 0.001). While echocardiography is a versatile tool in the complex geometry of the Ebstein heart, it has limitations. CMR offers a total overview and has the advantage of reliable volume assessment of both ventricles. Comprehensive evaluation of pediatric patients with EA may therefore require a synergistic implementation of echocardiography and CMR.

**Supplementary Information:**

The online version contains supplementary material available at 10.1007/s00246-022-02948-3.

## Introduction

Ebstein anomaly (EA) is a rare congenital heart disease occurring in 1–3 per 100,000 live births [1]. It is characterized by abnormal myocardial development of the right ventricle (RV) and embryonic delamination failure of the septal, inferior, and anterior leaflets of the tricuspid valve (TV). The displaced hinge points of the septal and inferior leaflet create a functional annulus anteriorly towards the right ventricular outflow tract. The critical distinguishing feature of EA is a displacement of the septal leaflet hinge point of ≥ 8 mm/m^2^ body surface area [2]. The orifice of the functional annulus can be more than tripled in area compared to normal. The plane of the annulus is often rotated > 30° [3].

The inlet portion of the RV is functionally integrated with the often dilated right atrium (RA) and is referred to as the atrialized RV (aRV). The anatomical atrioventricular orifice is usually significantly enlarged [4]. The trabecular and outlet portions of the RV constitute the functional RV (fRV). The size of the fRV can be more than doubled at both end-diastole and end-systole compared to the normal right ventricle [3, 4]. The base shows significant bulging and the apex is rounded. The function of the fRV is usually significantly decreased [3].

This complex geometry causes challenges in the assessment of RA and RV function. Nonetheless, assessment of anatomical and functional parameters is important for prognosis and planning of potential surgical interventions [5, 6]. Traditional indications for surgery include symptoms as fatigue, cyanosis in case of interatrial shunting, shortness of breath, or decreased exercise tolerance. Other indications for surgery include progressive fRV enlargement or fRV dysfunction and the onset of tachyarrhythmias [7]. The timing of surgery has evolved over the last decades and is now believed to be during childhood [8]. While patient selection begins with thorough imaging, it is essential to study the use of echocardiography and cardiac magnetic resonance (CMR) imaging for anatomical and functional assessment of children and adolescents with EA.

Echocardiography is the most used modality for diagnosis and serial follow-up of patients with EA, as it is widely available by the bedside and applicable to every age, generally without the need for anesthesia. However, due to the generally severely enlarged fRA and fRV, it can be difficult to obtain good views, which limits reliable functional assessment. CMR has become a complementary and comprehensive tool for accurate and reproducible assessment of fRV function and tissue characterization in EA [9, 10]. However, the anomalous geometry of EA may be difficult to incorporate in standardized imaging protocols. Moreover, children may find the relatively long imaging protocols in the bore of a CMR scanner challenging and may need sedation.

As both imaging techniques have unique limitations and strengths, combining the modalities could be synergistic. The goal of the current study was threefold: (1) to assess biventricular function by echocardiography and CMR in young patients with EA; (2) to compare EA severity scores calculated by echocardiography and CMR; and (3) to compare imaging parameters with clinical markers of heart failure.

## Material and Methods

### Study Participants

In this cross-sectional multicenter study, all consecutive pediatric patients (aged 8–17 years) with EA who underwent routine clinical assessment in one of the five participating Dutch academic hospitals between May 2017 and March 2019 were included. Patients with univentricular palliation, excluding the RV from circulation, were excluded. Patients had a medical history taken and underwent physical examination, electrocardiogram, cardiopulmonary exercise testing, echocardiography, and CMR.

In accordance with the ethical guidelines of the 1975 Declaration of Helsinki [11] the study protocol was assessed by the Medical Ethics Committee of the Erasmus Medical Center (protocol number MEC-2016-752) and by the institutional review boards of all participating centers. Written informed consent was obtained from the legal guardian(s) of all participants and from participants aged 12 or older.

### Echocardiography

All participants underwent transthoracic echocardiography, and standard subxiphoid, left parasternal long- and short-axis, apical, suprasternal notch, and right parasternal views were acquired. All studies were supervised by a senior pediatric cardiology member of the research team. Each study was performed at rest on a locally available machine*.* Three to five subsequent heart beats were recorded.

### Echocardiographic Parameters

All echocardiographic studies were analyzed in a single core lab by two cardiologists, L.G. performed the initial measurements and final values were obtained after consultation with L.K., an experienced cardiologist. The following echocardiographic parameters for assessment of biventricular dimensions and function were included: maximum peak flow velocities in both outflow tracts, the great arteries, and across the atrioventricular valves (Doppler E and A); maximal mitral and tricuspid annular plane systolic excursions on M-mode images (MAPSE and TAPSE, respectively); fRV fractional area change (fRV-FAC); left ventricle (LV) ejection fraction (LVEF) by modified Simpson method; tissue Doppler peak systolic myocardial velocity (s′) of the fRV (fRVs’) and LV (LVs’) free wall (FW) and peak systolic longitudinal strain (peak systolic strain) of the LV in four-, three- and two-chamber views and peak systolic strain of the fRV FW. The severity of the tricuspid regurgitation (TR) was graded 0 (no regurgitation), 1 (mild), 2 (moderate) and 3 (severe), using the approach as recommended by the American Society of Echocardiography [12]. These recommendations included color jet area, vena contracta width, density of continuous Doppler jet, and hepatic vein flow pattern.

Reference values of both MAPSE and TAPSE and fRVs’ and LVs’ in children are age- dependent. Therefore, results were compared to age-matched reference values [13, 14]. Values two or more standard deviations (SDs) below or above the mean value were considered reduced or increased, respectively. Feature tracking analysis was performed to obtain longitudinal strain of the endocardial wall, according to task force recommendations [15], using the offline TomTec Arena software (TTA2, TomTec Imaging Systems GmbH, Unterschleissheim, Germany) and results were compared to normal ranges in children [16].

**Fig. 1:**
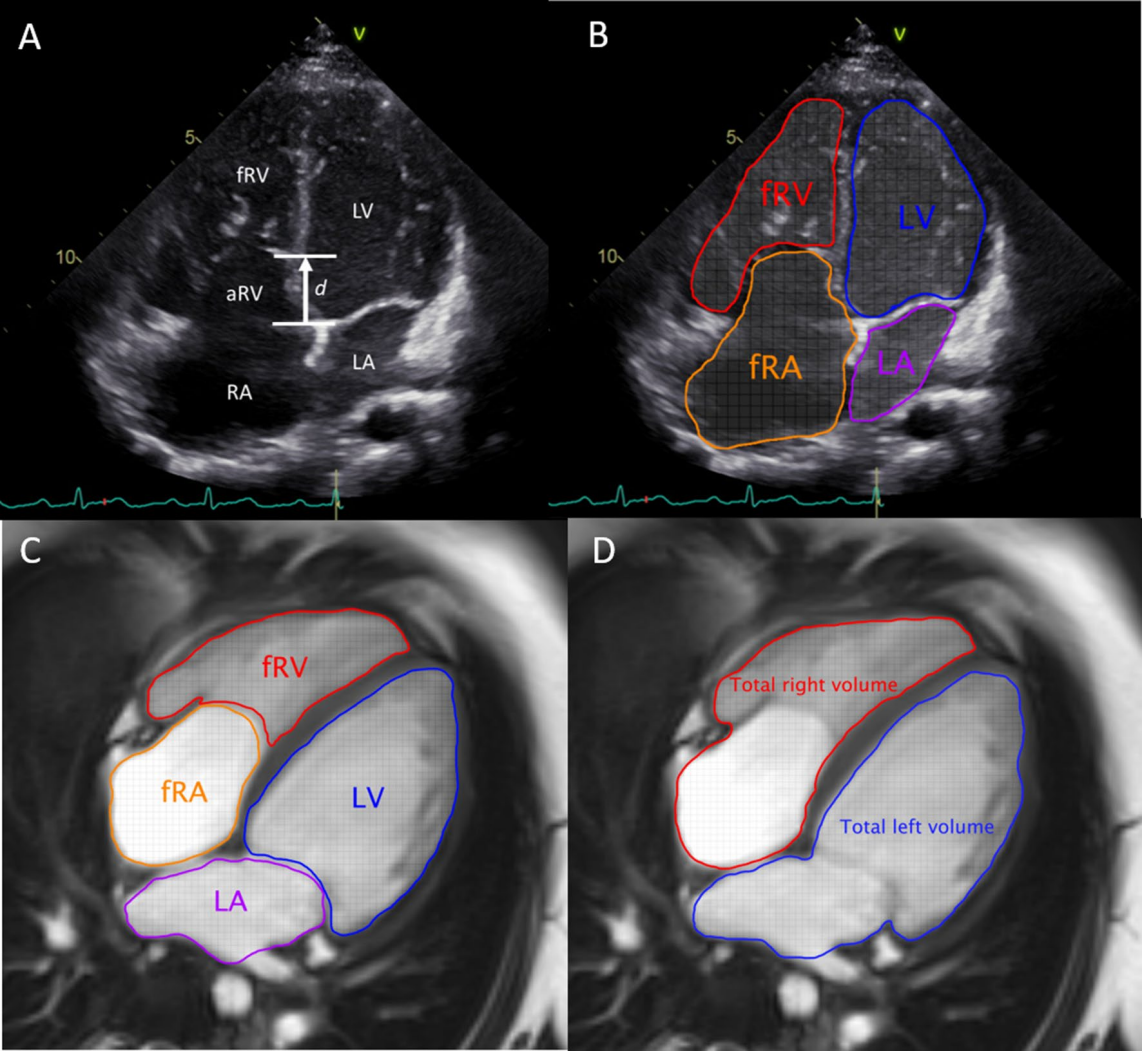
Measurement of the displacement index (**A**). Celermajer index by echocardiography (**B**). Overview of segmentation of Celermajer index (**C**) and total right/left volume ratio (**D**) on CMR. Note that actual ventricular volumes were obtained on a volumetric stack of short-axis slices. *CMR* cardiac magnetic resonance, *fRA* functional right atrium, *fRV* functional right ventricle, *d* distance, *LA* left atrium, *LV* left ventricle

The displacement index was measured in unrepaired patients with EA using the end-diastolic apical four-chamber view. The distance from the insertion of the anterior mitral valve leaflet to the displaced hinge point of the septal leaflet of the TV was measured (Fig. 1A) and indexed to the body surface area. The Celermajer index (CI), the ratio of the RA and aRV area to that of the fRV, left atrium (LA) and LV area, was calculated in unrepaired patients in a four-chamber view at end-diastole (Fig. 1B). The acquired values were classified into four grades: grade 1 (CI < 0.5), grade 2 (CI 0.5 to 0.99), grade 3 (CI 1 to 1.49), and grade 4 (CI > 1.5). [17] Higher grades indicate more severe EA.

### CMR Imaging

CMR was performed according to a standardized study protocol on locally available machines, all with 1.5-Tesla field strengths. Studies were performed by experienced technicians; no sedation was administered. All images were ECG-gated and obtained during breathhold. A multiphase, multislice volumetric data set was acquired using a fast 2-dimensional SSFP cine scan in short-axis orientation, covering the ventricles from base to apex. The following MRI acquisition parameters were used: TR 3.4, TE 1.4, flip angle 45°, and slice thickness 8 mm. Predefined FOV sizes were 36 × 36, with a matrix size of 224*192 for cine and 256 × 128 for phase-contrast (PC) flow. The number of reconstructed phases was limited to heart rate, with a minimum of 20 phases for SSFP and PC flow (median: 25). This data set was used to measure LV and fRV function. Standard four-, three- and two-chamber views with SSFP cine images were also obtained.

CMR flow quantification was performed during breathhold, in the ascending aorta, the pulmonary trunk and the tricuspid valve, with 2D fast PC sequences. Standardized velocity-encoded (VENC) settings were defined, although technicians could alter settings to reduce aliasing. TE was minimized, with a flip angle of 20°. Flow in the outflow tracts was measured on a phase-contrast sequence in an imaging plane perpendicular to the arterial jet at 10 mm from the valve’s orifice.

### CMR Parameters

All CMR studies were analyzed in a single core lab by two radiologists, initial measurements were performed by W.E., and final values were defined after consulting J.H., an experienced cardiothoracic radiologist. The following CMR parameters for assessment of biventricular dimensions and function were included on cine images: end-diastolic volume (EDV) and end-systolic volume (ESV) of the fRV and LV; EF and stroke volume (SV) of the fRV and LV; LV peak systolic strain in four-, three- and two-chamber view, and global fRV peak systolic strain and fRV FW peak systolic strain in the four-chamber view. TR was determined using a stepwise approach. Flow measurements were analyzed in Qflow (Medis, Leiden, The Netherlands). Qflow measurements were used if deemed of adequate quality. Indirect calculation of the TV regurgitation fraction (TV-RF) was performed using the fRV-SV and pulmonary artery (PA) forward and backward flow volume, using the following equation: ${\text{TVRF (\%)= [(fRVSV - PA forward flow - PA backflow))/(fRVSV)]}} \times {100}{\text{.}}$ Visual assessment on CINE images was used in cases with suboptimal Qflow measurements of the tricuspid and pulmonary valve. The most severe regurgitation was used to define TR grades comparable to echocardiography: 0 (no regurgitation), 1 (mild; 1–15%), 2 (moderate; > 20% 16–25%) and 3 (severe; > 26%) [18].

Volumetric data were derived after segmentation of the short-axis slices with QMass software (Medis, Leiden, The Netherlands). Trabeculae of the RV and LV were included in the volumes. Endocardial borders were manually traced. The aRV and fRV were defined according to Fratz et al. [4] Atrial volumes were determined on four- and two-chamber views, using the biplane area length method for the LA and the single plane Simpson's method of disks for the RA. Feature tracking analysis was used to obtain longitudinal strain of the endocardial wall, performed offline on long-axis slices using Qstrain. Results were compared to published age-dependent reference values [19, 20].

The tricuspid annulus displacement index was calculated as by echocardiography. CI was calculated as previously described by Cieplucha et al. [21] as the ratio of the end-diastolic volume of the RA and aRV to that of the summed end-diastolic volumes of the fRV, LA and LV (Fig. 1C). Total right/left-volume index was determined as proposed by Hösch et al. [22] End-diastolic volumes derived from CMR SSFP cine stacks were used for calculation of this index: (RA + aRV + fRV)/(LA + LV) (Fig. 1D).

### Cardiopulmonary Exercise Testing

Exercise tests were performed on a bicycle ergometer in the patient’s own outpatient clinic. Breath-by-breath gas exchange analysis was performed continuously to measure the respiratory parameters. Heart rate and rhythm were continuously monitored using 12-lead electrocardiography. Blood pressure was measured every 2 min. Oxygen saturation was measured using pulse oximetry. Patients were encouraged to exercise to exhaustion. A peak respiratory exchange rate of 1.05 during testing was considered a maximally performed test. Each test consisted of three phases: (1) a 1-min resting phase; (2) a phase with gradual or stepwise increments of 10–15 W/min; and (3) a 3-min recovery phase. Peak workload, oxygen pulse (O_2_-pulse) and maximal oxygen consumption (VO_2_max) were included as the percentage of predicted value (%Pred) for gender and age. The minute ventilation to carbon dioxide production slope (VE/VCO_2_ slope) was measured from the start of exercise until the respiratory compensation point.

### Statistical Analysis

Statistical analysis was performed using SPSS statistics version 25 (IBM, Armonk, New York, USA). Data is presented as mean ± standard deviation (SD) or median and interquartile range (IQR), based on normality of data. Normality of data was verified using QQ-plots. For correlation of echocardiography- and CMR-derived parameters and clinical parameters, the Spearman rank (rho) or Pearson correlation coefficient (*R*) were used, depending on normality of data. The CI grade and TR grade were compared using Cohen’s kappa. A Cohen’s kappa, *R* or rho value of ≥ 0.8 was classified as excellent, 0.60–0.79 as good, 0.40–0.59 as moderate, and < 0.40 as poor.

## Results

### Study Participants

**Fig. 2:**
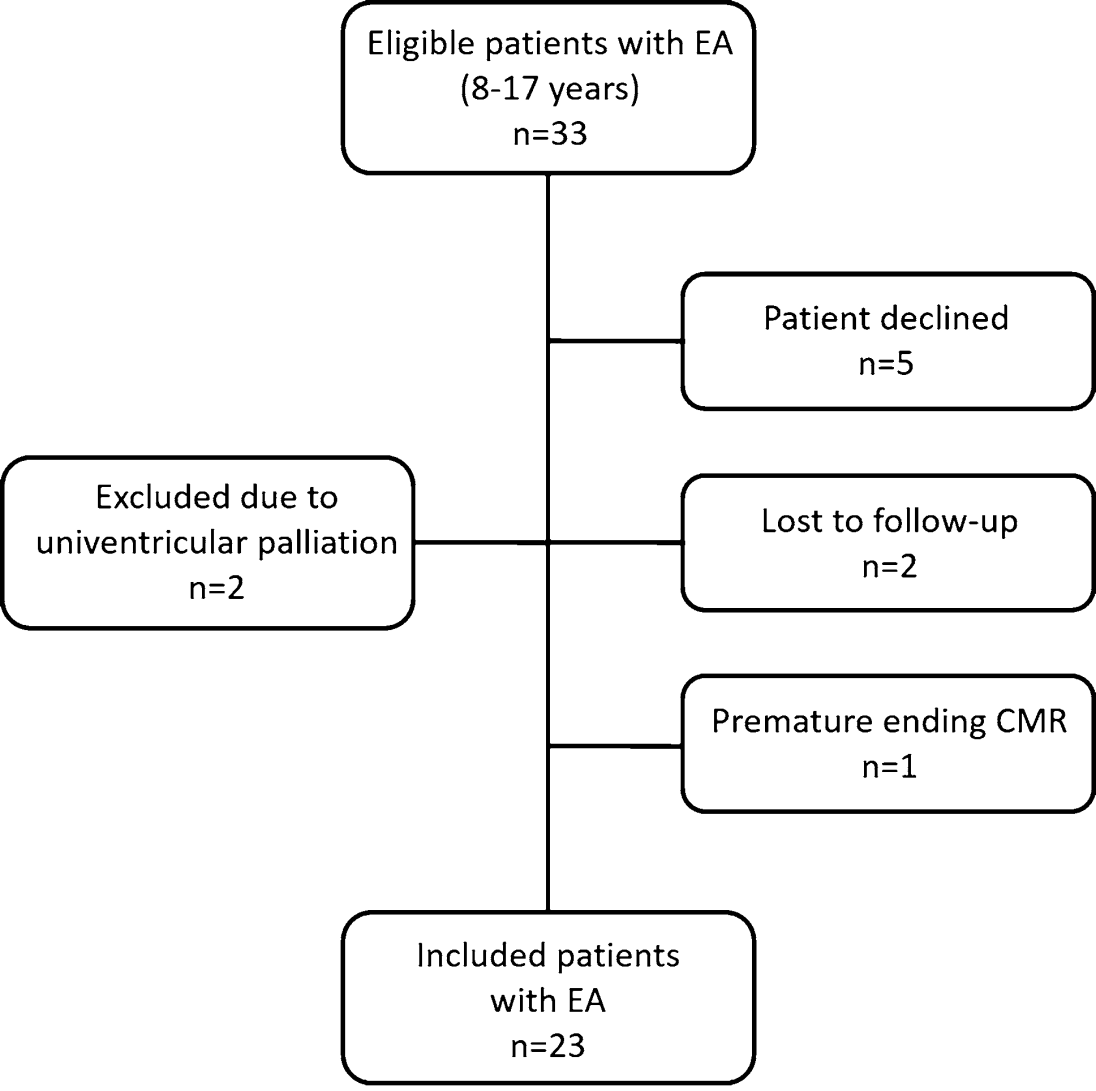
Study inclusion diagram. Two patients were lost to follow-up because of incorrect contact data. In one patient the CMR was ended prematurely due to anxiety

Twenty-three patients (8–17 years old) underwent both echocardiography and CMR (Fig. 2). The demographic characteristics and functional health status are shown in Table 1. TAPSE was derived in all patients with echocardiography, fRV-FAC and fRVs’ were acquired in 17 (74%) and 20 (87%) patients, respectively, and fRV FW peak systolic strain in 20 (87%) patients. Echocardiography-derived MAPSE was obtained in all patients. The LVEF and the peak systolic strain LV strain were obtained in seven (30%) and six (26%) patients, respectively. The feasibility of all predefined CMR-derived parameters was 100%, except for Qflow measurements of the tricuspid valve, which was feasible in only 8 patients (35%). The indirect measurement of TV-RF, using RVSV and Qflow of the pulmonary artery was used in the remaining cases. Echocardiography- and CMR-derived parameters for assessment of biventricular function and EA severity scores for each patient are shown in supplemental Table 2.

**Table 1:** Demographic characteristics and functional health status of all 23 participants

Biographical characteristics	*n* = 23
Male	9 (39)
Age in years	14 (12–15)
*Medical history*	
Relevant right sided co-morbidity	
Pulmonary atresia	2 (9)
Pulmonary stenosis	1 (4)
Relevant left sided co-morbidity	
LVNC-cardiomyopathy	1 (4)
Mitral valve dysplasia	1 (4)
Aortic coarctation	1 (4)
Previous TV repair*	5 (20)
VO_2_max (% predicted)	74 (57–80)
NYHA I	20 (87)
NYHA II	3 (13)

*One additional patient received surgical closure of an atrial septal defect type II, and one additional patient had an end-to-end coarctectomy, both did not have tricuspid valve surgery. Data as number and percentage or interquartile range between brackets

*LVNC* left-ventricular non-compaction, *TV* tricuspid valve, *VO*_*2*_*max* maximal oxygen uptake

### Right Ventricle

By echocardiography, it was often difficult to obtain good images of the fRV FW due to the balloon-shape of the dilated RV. Especially in the four-chamber view, the rounded apical part often bulged out of plane. Although the median fRV-FAC was 38% (33–42) (Table 2), and within the normal range, TAPSE and fRVs’ were reduced in 39% and 75% of our patients respectively. Median fRV FW peak systolic strain was reduced with − 23.5% (− 20.9 to − 28.9). TR was visually graded as mild, moderate or severe in nine, six and eight patients, respectively. For echocardiographic evaluation of fRV size, short-axis views seemed more illustrative than the standard four-chamber views. In the youngest patients, subxiphoid views were helpful.

**Table 2:** Functional parameters obtained with both imaging modalities

	Echocardiography *n* = 23	CMR *n* = 23
fRV-FAC (%), *n* = 17	38 (33–42)	–
fRVEDV (ml/m^2^)	–	82 (70–105)
fRVEF (%)	–	49 (36–58)
TR (*n*, %)		
Mild	9 (39)	9 (39)
Moderate	6 (26)	12 (52)
Severe	8 (35)	2 (9)
LVEDV (ml/m^2^)	–	77 (70–83)
LVEF (%)	–	58 (49–63)

*CMR* cardiac magnetic resonance, *EDV* end-diastolic volume, *EF* ejection fraction, *FAC* fractional area change, *fRV* functional portion of the right ventricle, *LV* left ventricle, *TR* tricuspid valve regurgitation

By CMR, median fRVEF was 49% (36–58). Both global and FW fRV peak systolic strain were reduced with − 13.8% (− 10.3 to − 18.0) and − 17.7% (− 11.1 to − 26.2), respectively. TR was graded as mild, moderate or severe in nine, twelve and two patients, respectively. Enlargement of the fRV was seen in 52% of the patients. The fRV-EDV was higher than the LV-EDV in 16 out of 23 patients (70%). The median total right/left-volume index in unrepaired EA patients was 1.5 (IQR 1.3–2.5).

Correlation between echocardiography- and CMR-derived parameters demonstrated an excellent correlation (rho = 0.812, *p* < 0.001) between fRV-FAC and fRVEF (Fig. 2D). Compared to fRVEF on CMR, fRV-FAC underestimates RV function. Both TAPSE (rho = − 0.336, *p* = 0.117) and fRVs’ (rho = − 0.413, *p* = 0.07) showed a non-significant, but inverse relation to fRVEF. Echocardiography-derived fRV FW peak systolic longitudinal strain showed no significant correlation to fRVEF (*r* = − 0.289, *p* = 0.217), nor to other RV function parameters Although CMR-derived fRV global peak systolic strain had a moderate association with fRVEF (rho = − 0.425, *p* = 0.043), fRV FW peak systolic strain did not (rho = − 0.196, *p* = 0.369).

### Left Ventricle

LV function and size were often difficult to assess with echocardiography. The LV focused four-chamber view was acquired in almost all patients, but the acquisition of reliable three- or two-chamber views was more challenging. The LV appeared rather compressed and less spherical, especially in the case of a high displacement index and abnormal interventricular septal motion. This substantially hampered reliable calculation of the LVEF by the modified Simpson method and adequate LV feature tracking. Therefore, MAPSE and LVs’ were assessed for evaluation of the LV long-axis function. MAPSE and LVs’ were reduced in 52% and 71% of the patients, respectively. In the left parasternal long-axis view, the LV outflow tract often appeared narrow due to severe septal bulging towards the LV cavity. However, no turbulence was seen by color Doppler and peak velocities up to only 1.36 m/s were calculated in left ventricular outflow tracts in standard five-chamber views.

By CMR, short-axis cine images for measuring LV volumes were of good quality in almost all (96%) cases. LVEF was (slightly) decreased in 17 cases (74%) and normal in the remaining cases. The median LV peak systolic longitudinal strain was reduced with − 18.8%. LV-EDV was reduced in seven (30%) patients.

Correlation between echocardiography- and CMR-derived parameters showed moderate correlation (rho = 0.500, *p* = 0.015) between MAPSE and LVEF. There were no significant correlations between MAPSE and CMR-derived LV global peak systolic strain (rho = − 0.280, *p* = 0.195), as well as between CMR-derived LV global peak systolic longitudinal strain and LVEF (rho = − 0.170, *p* = 0.438). There was no correlation between echocardiography-derived LVs’ and CMR-derived LVEF either (rho = 0.148, *p* = 0.558).

### Comparison of Severity Scores

**Fig. 3:**
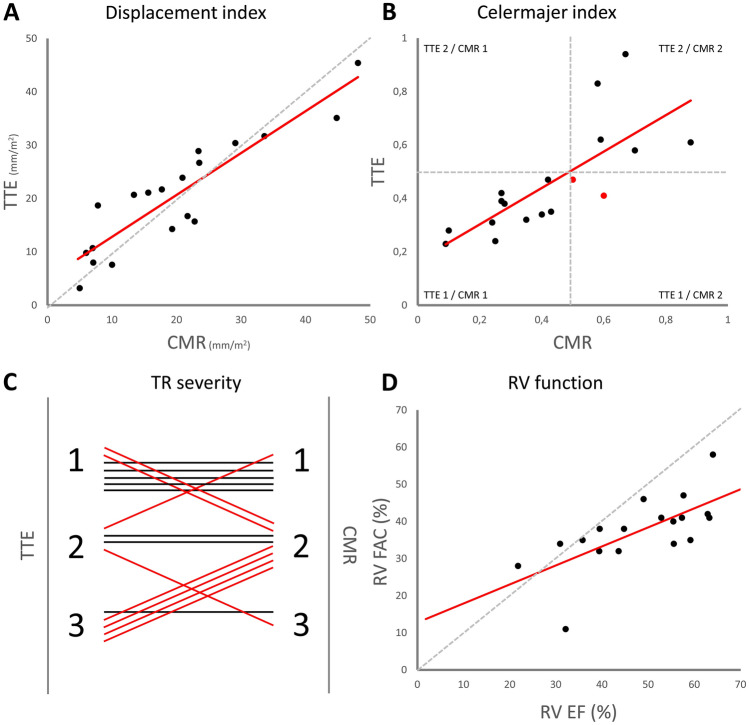
Correlations between echocardiography- and CMR-derived Ebstein anomaly severity scores: **A** Displacement index, **B** Celermajer index, **C** TR severity and **D** RV function. *CMR* cardiac magnetic resonance, *RV* right ventricular, *TR* tricuspid regurgitation, *TTE* transthoracic echocardiography

There was excellent correlation (rho = 0.853, *p* < 0.001) between the displacement index measured by echocardiography (20.9 mm/m^2^, 13.4–29.3) and CMR (20.1 mm/m^2^, 9.5–24.9) (Fig. 3A). Correlation between the CI by echocardiography (0.40, 0.32–0.59) and CMR (0.41, 0.26–0.59) was also excellent (rho = 0.837, *p* < 0.001) (Fig. 3B). Translated to grades, the CI showed similar agreement, (Cohen’s kappa 0.753, *p* < 0.001). TR severity was graded lower by CMR compared to echocardiography in five patients, and higher in two patients (Fig. 3C). Agreement between both parameters was moderate (Cohen’s kappa 0.425, *p* < 0.001).

### Comparison of Functional Parameters with Clinical Markers of Heart Failure

Sixteen patients (70%) performed cardiopulmonary maximum exercise testing until exhaustion. There was good correlation between the echocardiography- or CMR-derived displacement index and the VE/VCO_2_ slope (rho = 0.703 and rho = 0.792, respectively). There was no significant correlation between echocardiography- or CMR-derived functional parameters and results from cardiopulmonary exercise testing (Supplemental Table 1).

Most of the 23 patients (87%) were categorized as New York Heart Association (NYHA) functional class I, with only three patients being categorized as functional class II (13%). In the first patient with NYHA functional class II, higher EA severity scores were found, the TR was scored moderate to severe by echocardiography and the CMR-derived RVEF was severely reduced at 31%. The second and third patients with NYHA functional class II, however, showed low EA severity scores, less TR and the RVEF was within normal range. There were no significant differences between patients in NYHA functional class I or II regarding the obtained imaging parameters.

## Discussion

This study evaluates clinical use of echocardiography and CMR in young patients with EA. By echocardiography, it was often difficult to acquire images encompassing the entire fRV. Especially in four-chamber views, the rounded apical part often bulged out of plane. Left ventricular functional assessment by echocardiography was challenging as well, as the LV appeared rather compressed and less spherical, especially in the case of a severe displacement index and abnormal interventricular septal motion. By CMR, assessment of both fRV and LV volumes and function were feasible, while volumetric assessment was often suboptimal with echocardiography. Correlation between echocardiography-derived fRV-FAC and CMR-derived fRVEF was excellent, although echocardiography underestimates RV function. MAPSE and CMR-derived LVEF had moderate correlation. The correlation between echocardiography- and CMR-derived TR severity was only moderate, while correlation between the severity indices was excellent. Finally, there was a good correlation between the echocardiography- and CMR-derived displacement index and the VE/VCO_2_ slope.

In general, CMR-derived fRVEF is considered the gold standard for functional assessment of the fRV. Nevertheless, implementation and serial follow-up with CMR can be challenging, especially in younger patients. Although it underestimates RV function, echocardiography-derived fRV-FAC showed good correlation with CMR-derived fRVEF. However, echocardiographic acquisition of proper fRV views can be difficult in patients with EA, and volumetric parameters were therefore not feasible with echocardiography. Alternative functional parameters, such as TAPSE and RVs’ are often used for qualitative assessment of fRV longitudinal function. TAPSE showed an inverse relation with fRVEF, contradictory to expected findings. It should be considered that TAPSE and RVs’ might overestimate functional RV performance, as the contribution of tricuspid annular movement to the functional RV may be decreased due to altered geometry in EA. CMR-derived fRV global peak systolic strain showed a significant association with fRVEF. It would be of great interest to further investigate if reduced fRV global peak systolic strain can predict deterioration of fRV function.

Abnormal interventricular septal motion made LV assessment by echocardiography demanding. Below the hinge point of the TV septal leaflet, the septal wall showed leftward diastolic displacement, altering LV geometry and reducing cavity size. This was not counterbalanced by hyperactivity of the LV FW; MAPSE and LVs’ were reduced in 52% and 71% of patients, respectively.

Grading TR severity by echocardiography is difficult. The current recommendations of the American Society of Echocardiography include jet characteristics, vena contracta width, hepatic venous backflow, and signal density of the regurgitation jet, but these are hardly applicable in patients with EA. Due to the anterior rotation of the functional TV annulus towards the RVOT, the TR jet cannot be fully appreciated in one single plane. Furthermore, the TR usually comprises a various number of jets originating from both coaptation defects and leaflet fenestrations, which limits accurate assessment of the jet and may result in underestimation of TR severity. The maximal TR jet velocity might be lower than expected due to the enlarged RA and decreased fRV function. Inferior caval vein enlargement or hepatic vein systolic flow reversal is seen infrequently because of the presence of the severely enlarged RA.

TR quantification by CMR velocity mapping was also challenging, as only 35% of cases were deemed of adequate quality. Velocity mapping of the TR is hindered by the multiple directions of the regurgitation jets, which are difficult to encompass in one plane. Moreover, jets may change direction throughout the cardiac cycle because of systolic motion of the enlarged RV. The indirect quantification using velocity mapping of the pulmonary valve and fRV-SV, was feasible in all cases. Nevertheless, measurement is indirect and may suffer from difficulties in acquisition. In cases of suboptimal velocity-encoding parameters or incorrect positioning and/or angulation, flow characteristics will be misinterpreted. In future studies, 4D flow assessment of valves may offer a solution, providing datasets with multidimensional possibilities, especially in complex multidirectional flow patterns of the TV in EA [23]. Although promising, studies on 4D flow in patients with EA are limited to case reports [24].

Grading EA severity has always been challenging. Several echocardiographic severity scores have been proposed, of which the Carpentier classification and the CI are the most used. The Carpentier classification, proposed in 1988 [25], incorporates fRV size and anatomic features of the TV anterior leaflet, and distinguishes four severity types. This classification has restrictions because the functional severity is determined by various components and a combination of types can be seen. In 1994, Celermajer et al. [17] described an alternative echocardiographic grading score, initially for neonates, in which the area ratio between the sum of the RA and aRV and the sum of fRV, LA and LV were calculated. Although this classification was developed for echocardiography, its value in CMR has been subject to studies. Cieplucha et al. [21] compared the echocardiography- and CMR-derived CI and found a moderate agreement, as echocardiography usually overestimated but rarely underestimated EA severity. In our population, the correlation of the CI between the two imaging modalities was excellent. In two patients, the CI was higher by CMR as compared to echocardiography, although the absolute difference was small. Calculation of the CI by echocardiography requires acquisition of excellent four-chamber views, in which the FW of the fRV is completely visible and both the LA and LV are fully appreciated. In our experience, obtaining such four-chamber views can be problematic. Scoring EA severity by CMR-derived CI overcomes this difficulty.

By CMR, the median right/left-volume index in patients with unrepaired EA was 1.5, with only six patients scoring > 2.5. Hösch et al. [22] were the first to propose this simplified CMR measurement for scoring EA severity. They found a relatively high mean total right/left-volume index of 2.6 ± 1.7 (normal value 1.1 ± 0.1) in their EA population. This volume index correlated with almost all clinical biomarkers of heart failure and was considered a more accurate assessment of disease severity than previously described scoring systems. The difference in severity scores between the current population and that of Hösch et al. might be explained by the mean age of the study groups: 26 ± 14 years for Hösch et al. compared to 13 ± 3 years in our cohort. Younger patients may have less severe TR with less dilatation of the aRV, resulting in lower total right volumes and consequently lower severity scores.

There was good correlation between the echocardiography- and CMR-derived displacement index and VE/VCO_2_ slope. There were no other significant correlations between echocardiography- or CMR-derived parameters and results from cardiopulmonary exercise testing. This could be explained by the fact that the young EA population showed relatively good biventricular function and mild EA severity scores. Hypothetically, in patients with significantly reduced biventricular function and more severe EA, a better correlation with the functional status might be seen. This emphasizes the relevance of additional pediatric studies, in larger populations, to further explore the relations between imaging parameters, functional status, and other outcome measures.

### Strengths and Limitations

This is the first combined echocardiography and CMR study to specifically target young EA patients (< 18 years old). The multicenter recruitment of consecutive patients, the broad age range and the fact that both surgical and non-surgical patients were included, introduces bias in the results. Moreover, clinical severity (e.g., NYHA class) was limited in the study population, impeding translation of results to more severe states of EA. However, including a wide spectrum of patients with EA over multiple academic centers improves the generalizability of our results in the general pediatric EA population. Although the sample size is relatively large for youngsters with EA, the number of patients limits extensive statistical analysis and decreases statistical power. Although an intra- and interobserver analysis could further elucidate the role of CMR and echocardiography in patients with EA, variability of implemented parameters was beyond the scope of this study. The complex anatomy of the RV causes continued debate on the optimal slice orientation for RV volumetric assessment with CMR. While an axial slice orientation might offer an alternative, short-axis image stacks are non-inferior to axial slices [26–28]. Imaging protocols were kept concise to reduce scan duration, therefore only short-axis images were obtained. Comparability of strain values to reference standards may be hindered by differences between specific software packages and methods (i.e., endo- vs. mid- or epicardial wall strain) [29]. However, methods of the current study were made comparable for both CMR and echocardiography, both implementing tracking of the endocardial border, thereby reducing bias.

## Conclusion

**Fig. 4:**
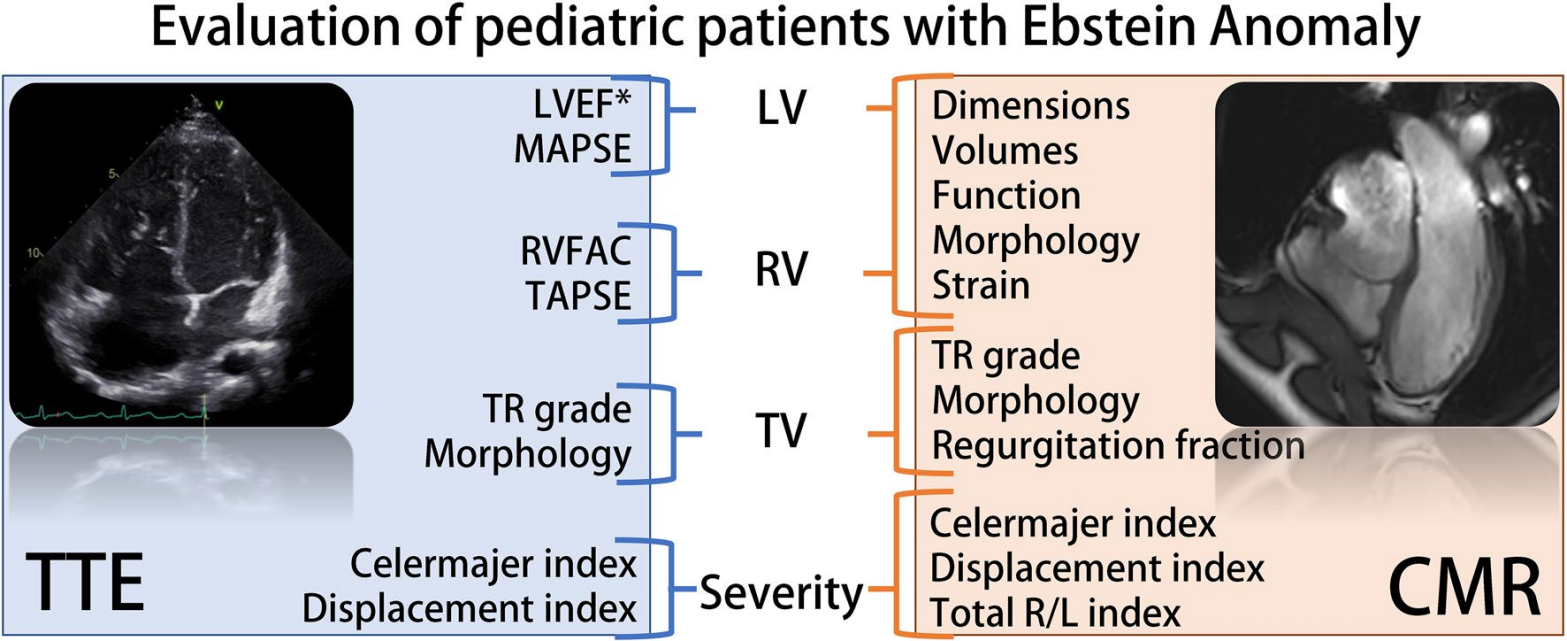
Central illustration. Recommended parameters for comprehensive evaluation of Ebstein Anomaly in pediatric patients. *LVEF: left ventricular ejection fraction with TTE only in selected cases. *CMR* cardiac magnetic resonance, *EF* ejection fraction; *MAPSE* maximal mitral annular plane systolic excursion, *RVFAC* right ventricular fractional area change, *TR* tricuspid regurgitation, *TAPSE* maximal tricuspid annular plane systolic excursion, *TTE* transthoracic echocardiography; total R/L index, total right/left volume index

With the approach of childhood surgery in patients with EA, thorough imaging has become crucial for patient selection. The complex geometry of the Ebstein heart hinders reliable echocardiographic assessment of biventricular dimensions and function. While severity scores are comparable between imaging techniques, reliable volumetric and functional assessment requires CMR (Fig. 4, central illustration). Comprehensive evaluation of pediatric patients with EA may therefore require a synergistic implementation of echocardiography and CMR.

## Supplementary Information

Below is the link to the electronic supplementary material.

Supplementary file1 (DOCX 19 kb)
