# Supplementary material for: Comprehensive Evaluation of Pediatric Patients with Ebstein Anomaly Requires Both Echocardiography and Cardiac Magnetic Resonance Imaging
Source: Pediatr Cardiol. 2022 Jun 21;44(1):75–85. doi: 10.1007/s00246-022-02948-3 (PMC9852135; doi:10.1007/s00246-022-02948-3)
Supplement: Supplementary file 1 — Supplementary file1 (DOCX 19 kb) [file 246_2022_2948_MOESM1_ESM.docx]

**SUPPLEMENTAL MATERIAL**

**Supplemental table 1.** Correlation between echocardiography- and CMR-derived functional parameters, Ebstein anomaly severity scores and cardiopulmonary exercise testing results.

| Parameters | Peak Workload  (%Pred) | O_2_-pulse  (%Pred) | VO_2_max  (%Pred) | VE/VCO_2_  slope |
| --- | --- | --- | --- | --- |
|  | rho p | rho p | rho p | rho p |
| CMR Displacement index | -0.077 0.812 | -0.249 0.487 | -0.166 0.607 | **0.792 0.011** |
| TTE Displacement index | 0.047 0.878 | -0.009 0.980 | -0.115 0.722 | **0.703 0.023** |
| CMR Celermajer index | 0.312 0.324 | -0.292 0.413 | -0.138 0.670 | 0.400 0.286 |
| TTE Celermajer index | 0.282 0.374 | -0.028 0.938 | 0.075 0.817 | 0.519 0.152 |
| TTE fRV-FAC | -0.196 0.501 | 0.013 0.968 | -0.009 0.976 | -0.341 0.304 |
| CMR fRVEF | -0.176 0.515 | 0.092 0.764 | -0.120 0.671 | -0.323 0.306 |
| CMR LVEF | -0.119 0.660 | 0.316 0.292 | 0.141 0.617 | -0.319 0.312 |

*O2-pulse, oxygen-pulse; rho, Spearman rank; TTE, transthoracic echocardiography; VCO_2_, carbon dioxide production; VE, minute ventilation; %Pred, percentage of predicted value for gender and age. For other abbreviations, see table 1 and 2.*

**Supplemental table 2**. Echocardiography(^e^)- and CMR(^c^)-derived parameter for assessment of biventricular function, and Ebstein anomaly severity scores of all 23 participants

| Pt | Gender  M/F | Age  (years) | DI^e^  mm/m^2^ | CI^e^  grade | Total^c^  R/L  VI | TR^e^  grade | fRV^e^  FAC  (%) | fRV^c^  EDV  ml/m^2^ | fRV^c^  EF  (%) | LV^c^  EDV  ml/m^2^ | LV^c^  EF  (%) |
| --- | --- | --- | --- | --- | --- | --- | --- | --- | --- | --- | --- |
| 1 | F | 15 | 23.9 | 2 | 2.55 | 1 | 35 | 95 | 36 | 77 | 53 |
| 2 | M | 13 | - | - | - | 1 | 42 | 80 | 63 | 75 | 63 |
| 3 | F | 15 | 8.3 | 1 | 1.13 | 3 | nf | 70 | 64 | 66 | 58 |
| 4 | F | 12 | - | - | - | 3 | 47 | 99 | 58 | 82 | 49 |
| 5 | M | 9 | 16.7 | 1 | .89 | 1 | 38 | 53 | 45 | 84 | 45 |
| 6 | M | 16 | 28.9 | 2 | 3.17 | 3 | 32 | 117 | 39 | 70 | 63 |
| 7 | M | 15 | - | - | - | 3 | nf | 209 | 25 | 71 | 69 |
| 8 | M | 16 | 35.1 | 2 | 2.20 | 2 | nf | 84 | 25 | 60 | 62 |
| 9 | F | 8 | 45.4 | 2 | 2.51 | 2 | nf | 80 | 51 | 74 | 46 |
| 10 | F | 17 | 18.7 | 1 | 1.04 | 1 | 38 | 89 | 39 | 81 | 50 |
| 11 | F | 14 | 21.1 | 1 | 1.49 | 2 | 41 | 67 | 57 | 59 | 65 |
| 12 | F | 12 | 31.7 | 1 | 2.77 | 3 | 28 | 108 | 22 | 45 | 40 |
| 13 | F | 10 | 15.7 | 1 | 1.73 | 3 | 40 | 44 | 55 | 48 | 41 |
| 14 | F | 16 | 30.4 | 1 | 1.46 | 2 | 41 | 72 | 63 | 75 | 66 |
| 15 | M | 12 | 14.3 | 2 | 2.95 | 3 | 34 | 134 | 31 | 81 | 55 |
| 16 | F | 13 | - | - | - | 2 | 11 | 137 | 32 | 83 | 61 |
| 17 | F | 15 | 8.0 | 1 | 1.45 | 2 | 58 | 82 | 64 | 90 | 56 |
| 18 | M | 11 | - | - | - | 1 | nf | 105 | 47 | 84 | 58 |
| 19 | F | 17 | 26.7 | 1 | 1.67 | 1 | 32 | 81 | 44 | 76 | 63 |
| 20 | M | 14 | 20.7 | 1 | 1.36 | 1 | 41 | 99 | 53 | 91 | 59 |
| 21 | M | 15 | 21.7 | 1 | 1.38 | 1 | 46 | 65 | 49 | 78 | 61 |
| 22 | F | 12 | 9.8 | 1 | 1.16 | 3 | 34 | 78 | 56 | 91 | 58 |
| 23 | F | 10 | 10.7 | 1 | .80 | 1 | 35 | 44 | 59 | 78 | 48 |

*CI, Celermajer index; DI, displacement index; EDV, end-diastolic volume; EF, ejection fraction; ESV, end-systolic volume; F, female; FAC, fractional area change; fRV, functional portion of the right ventricle; LV, left ventricle; M, male; nf, not feasible; Pt, patient; Total R/L VI, total right/left volume index; TR, tricuspid regurgitation. Displacement index and severity scores were only calculated in non-surgical patients.*
